# Identification and Validation of Immune Cells and Hub Genes in Gastric Cancer Microenvironment

**DOI:** 10.1155/2022/8639323

**Published:** 2022-04-05

**Authors:** Huan Wang, Jianfang Rong, Qiaoyun Zhao, Conghua Song, Rulin Zhao, Sihai Chen, Yong Xie

**Affiliations:** ^1^The First Affiliated Hospital of Nanchang University, Nanchang, 330006 Jiangxi Province, China; ^2^Medical College of Nanchang University, Nanchang, 330006 Jiangxi Province, China

## Abstract

Gastric cancer (GC) is the most common malignant tumor in the digestive system, traditional radiotherapy and chemotherapy are not effective for some patients. The research progress of immunotherapy seems to provide a new way for treatment. However, it is still urgent to predict immunotherapy biomarkers and determine novel therapeutic targets. In this study, the gene expression profiles and clinical data of 407 stomach adenocarcinoma (STAD) patients were downloaded from The Cancer Genome Atlas (TCGA) portal, and the abundance ratio of immune cells in each sample was obtained via the “Cell Type Identification by Estimating Relative Subsets of RNA Transcripts (CIBERSORT)” algorithm. Five immune cells were obtained as a result of abundance comparison, and 295 immune-related genes were obtained through differential gene analysis. Enrichment, protein interaction, and module analysis were performed on these genes. We identified five immune cells associated with infiltration and 20 hub genes, of which five genes were correlated with overall survival. Finally, we used Real-time PCR (RT-PCR) to detect the expression differences of the five hub genes in 18 pairs of GC and adjacent tissues. This research not only provides cellular and gene targets for immunotherapy of GC but also provides new ideas for researchers to explore immunotherapy for various tumors.

## 1. Introduction

As a common malignant tumor, the GC has a high incidence of concealment and a high recurrence rate, which is the second cause of cancer death after lung cancer [1]. Approximately 990,000 people are diagnosed with GC each year worldwide, and most patients are already in a stage of local progression at the time of diagnosis, resulting in high mortality [2]. Traditional treatments such as chemotherapy, radiotherapy, and surgery are difficult for most patients with advanced GC to completely remove the tumor, and the 5-year survival rate of advanced GC treated with traditional methods is always on the low side [3, 4].

In recent years, with the understanding of tumor microenvironment and immune targets, immunotherapy has gradually become a new therapeutic method. Immunotherapy can reduce the pain of patients, improve the quality of life, and even prolong the survival time. Tumor immunotherapy mainly uses the body's natural defense mechanism to kill tumor cells, thereby enhancing antitumor immunity [5, 6]. At present, tumor immunotherapy mainly includes immune checkpoint inhibitors, adoptive cellular immunity, and immune vaccines. Immunotherapy is a new type of antitumor therapy, which has achieved certain results in the treatment of GC [7–9]. However, due to the complexity of human immune mechanisms, tumor-induced immune escape is a widespread phenomenon. There are still many problems that need to be addressed in GC immunotherapy, especially in predicting immunotherapy biomarkers and finding new effective therapeutic targets.

Cancer immunotherapy mainly cooperates with some important proteins to enhance or restore the function of immune cells in the tumor microenvironment. Therefore, we first studied the immune cells related to the degree of infiltration in STAD, then investigated the genes that are crucial to the level of infiltration of immune cells, and performed experiments to verify the results. Our study provides ideas and clues for the immunotherapy of STAD, and the identified cells and genes can be considered biomarkers for the prognosis or target of STAD therapy. In addition, this study also provides a new way for immunotherapy researchers to explore cellular and gene targets of immunotherapy.

## 2. Materials and Methods

### 2.1. Data Source and Preprocessing

**Figure 1:**
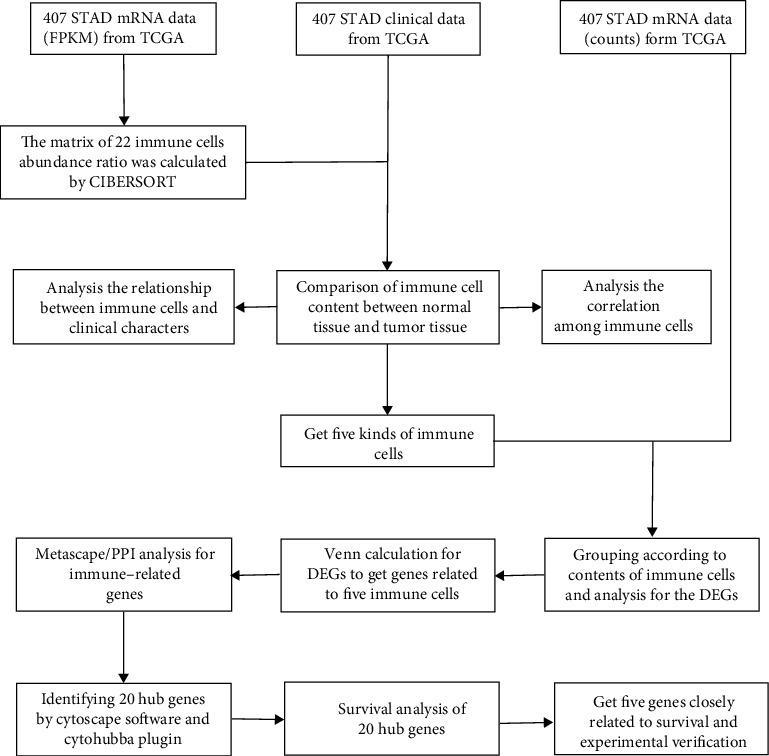
Flowchart of data acquisition and analysis process. TCGA: The Cancer Genome Atlas (https://portal.gdc.cancer.gov/). FPKM and counts are two different mRNA data formats in TCGA databases. CIBERSORT is a network tool that uses gene expression data to estimate the abundance ratio of member cell types in a mixed cell population. DEGs: differentially expressed genes. Metascape is a web-based portal designed to provide a comprehensive gene list annotation and analysis resource for experimental biologists. PPI: protein-protein interactions. Cytoscape is a network processing software, and the Cytohubba is a plugin in Cytoscape.

Download RNA-Seq gene expression profiles of 407 STAD patients (including 375 tumor samples and 32 normal samples) from the TCGA database, including FPKM and COUNT formats. Clinical data such as gender, age, tumor grade, clinical stage, and survival time were downloaded from the Genomic Data Commons (GDC) which was retrieved from the TCGA (https://tcga-data.nci.nih.gov/tcga/) database. Then, the R software was used to extract and sort the data to obtain the gene expression matrix and clinical data. This was followed by an analysis, and all the analytical processes are shown in Figure 1.

### 2.2. Identifying Immune-Related Cells

The CIBERSORT (https://cibersort.stanford.edu/) is an analytical tool developed by Newman that uses gene expression data to estimate the abundance ratio of member cell types in a mixed cell population [10]. To quantify the proportion of immune cells in STAD specimens, we used the CIBERSORT method and the LM22 gene signature [10]; the latter contains 547 genes, which can be highly sensitive and specific for the recognition of 22 human hematopoietic cell phenotypes (including B cells, T cells, natural killer cells, macrophages, dendritic cells, and myeloid subpopulations). The CIBERSORT uses the Monte Carlo sampling to derive a *P* value for the deconvolution of each sample, providing a measure of confidence in the results. At the threshold of *P* < 0.05, the results of the inferred part of the immune cell population produced by the CIBERSORT were considered accurate [11]. 164 samples (including 153 tumor samples and 11 normal samples) were selected by *P* < 0.05 for subsequent analysis. The Wilcoxon test was used to analyze the difference in the proportion of 22 immune cells in tumor samples and normal samples, and the cells with a significantly higher infiltration degree in tumor samples were identified as immune-related cells.

### 2.3. Clinical Relationship with Immune-Related Cells

Combined with the immune cell abundance ratio and clinical characteristics of 153 tumor samples, the relationship between immune cell abundance ratio and tumor grade, clinical stage, T-stage, and N-stage was analyzed by the independent samples *t*-test.

### 2.4. Identifying Immune-Related Genes

The STAD samples were grouped (high abundance group and low abundance group) according to the median abundance of the five immune cells identified in Section 2.2, by using the edgeR R software package and controlling ∣ log FC  | >1 and *P* < 0.05 to identify the differentially expressed genes (DEGs). An R package Venn diagram was used to generate these immune-related genes.

### 2.5. Enrichment Analysis of Immune-Related Genes

In order to elucidate the potential gene functional annotation and pathway enrichment associated with the 295 DEGs, Gene Ontology (GO) [12, 13] terms and Kyoto Encyclopedia of Genes and Genomes (KEG) [14–16] pathways were performed using the Metascape (http://metascape.org) [17]. These genes were assigned to functional groups according to molecular functions, biological processes, and specific pathways.

### 2.6. Protein-Protein Interaction Network Construction, Hub Genes, and Module Analysis

To assess the interactions among DEGs, the 295 immune-related genes were submitted to the String database (https://string-db.org/), a network tool for studying protein-protein interactions (PPI), and the comprehensive score was set to ≥0.4 [18]. An interactive network consisting of 148 nodes and 142 edges was obtained. The network was reconstructed by Cytoscape software (cytoscape.org) and a module analysis of the network was performed using the “MCODE” plugin [19] to search subnetworks of the PPI network. At the same time, important nodes in the network were predicted by the “Cytohubba” plugin [20], and the top 20 genes generated by Maximal Clique Centrality (MCC) were screened and identified as hub genes. Finally, we selected the module with the highest score from MCODE for enrichment analysis through the Metascape.

### 2.7. Survival Analyses of Hub Genes

Overall survival analyses of hub genes were performed using the GEPIA2 [21](http://gepia2.cancer-pku.cn/).

### 2.8. Patients and Tissue Specimens

Human gastric tissue samples (18 pairs of GC and adjacent samples) were collected from GC patients who underwent gastrectomy at the First Affiliated Hospital of Nanchang University. The diagnoses of GC were confirmed based on histology. All subjects provided informed consent for obtaining the study specimens. The study protocol was approved by the Clinical Research Ethics Committee of the First Affiliated Hospital of Nanchang University. All included cases were recorded in the Human Genetic Resources Center of the First Affiliated Hospital of Nanchang University.

### 2.9. Real-Time Quantitative PCR Analysis of the Five Hub Genes

TRIzol (Invitrogen) was then used to extract total tissue RNA, after which SYBR® Premix Ex Taq (TaKaRa) was used for RT–PCR. The primers used for the detection of human specimens are as follows: *β*-actin forward primer 5′-TGACGTGGACATCCGCAAAG-3′ and reverse primer 5′-CTGGAAGGTGGACAGCGAGG-3′; ADRA1B, forward primer 5′-CTTTCACGAGGACACCCTTAGC-3′ and reverse primer 5′-GCCCAACGTCTTAGCTGCTT-3′; BRS3 forward primer 5′-CTGCGTCTGGATCGTGTCTAT-3′ and reverse primer 5′-AGGGTCCTAGCAATCAAGGAAT-3′; CALCA, forward primer 5′-AAGCGGTGCGGTAATCTGAG-3′ and reverse primer 5′-GGGGAACGTGTGAAACTTGTTG-3′; CALCR forward primer 5′-CCTATCCAACAATAGAGCCCAAG-3′ and reverse primer 5′-TGCATTCGGTCATAGCATTTGTA-3′; OPRD1 forward primer 5′-CGTCCGGTACACTAAGATGAAGA-3′ and reverse primer 5′-GCCACGTCTCCATCAGGTA-3′. Student's *t*-test was used for comparison between the two groups. *P* < 0.05 was considered statistically significant.

## 3. Results

### 3.1. Data Source and Preprocessing

The RNA-Seq (including FPKM and counts) and clinical data of the 407 patients with STAD were obtained from the TCGA. Figure 1 flowchart shows the process of data acquisition and subsequent analysis.

### 3.2. Identifying Immune Cells in GC

**Figure 2:**
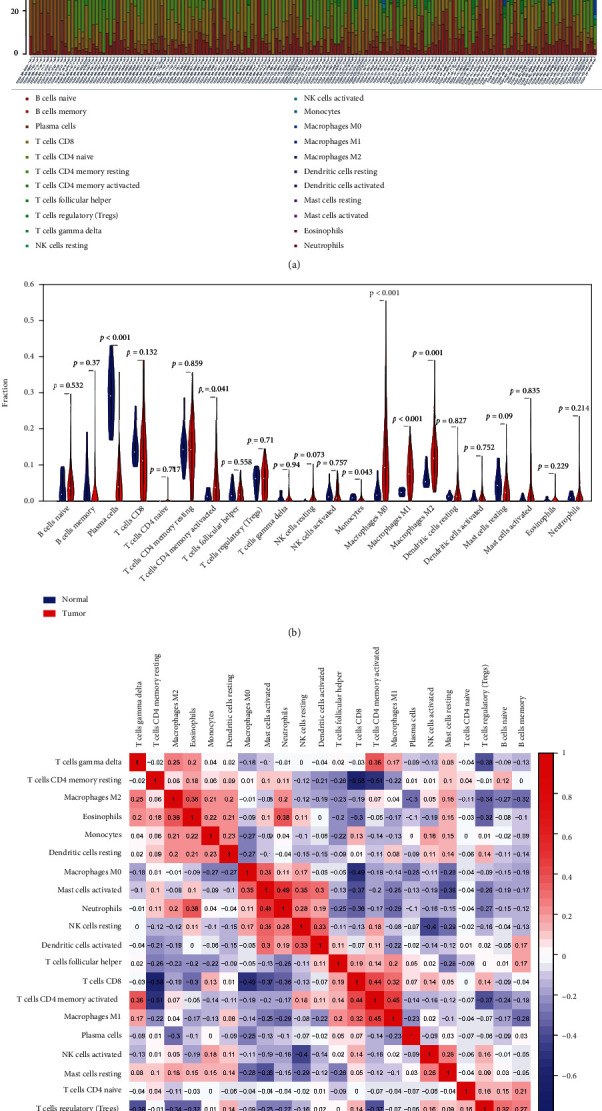
Identifying immune cells in GC. (a) The abundance ratio of immune cells in 164 samples. Each column represents a sample, and different colors and heights of each column represent the abundance ratio of immune cells in the sample. (b) Abundance ratio of 22 immune cells in cancer (*n* = 153) and normal (*n* = 11) samples. Blue represents normal samples, red represents tumor samples. (c) The relationship between abundance ratios of 22 immune cells. The value represents the relevant value. Red represents positive correlation, blue represents negative correlation.

By using the CIBERSORT, the abundance ratio of 22 immune cells in 164 STAD samples and the difference of the abundance ratios in cancer and normal samples were analyzed, as shown in Figures 2(a) and 2(b). The abundance ratio of plasma cell in normal samples (*n* = 11) was significantly higher than in cancer tissues (*n* = 153), while the contents of T cell CD4 memory activated, monocytes, macrophages M0, macrophages M1, and macrophages M2 in normal samples were significantly lower than that of tumor tissues. Besides, we also analyzed the correlation between the 22 immune cells. As shown in Figure 2(c), T cell CD4 memory resting was negatively correlated with T cell CD8 and T cell CD4 memory activated, while neutrophils and mast cells activated were significantly correlated. From the above results, it can be seen that the abundance ratios of T cell CD4 memory activated, monocytes, macrophages M0, macrophages M1, and macrophages M2 in GC were significantly higher than that of normal tissue.

### 3.3. Clinical Relationship with Immune-Related Cells

**Figure 3:**
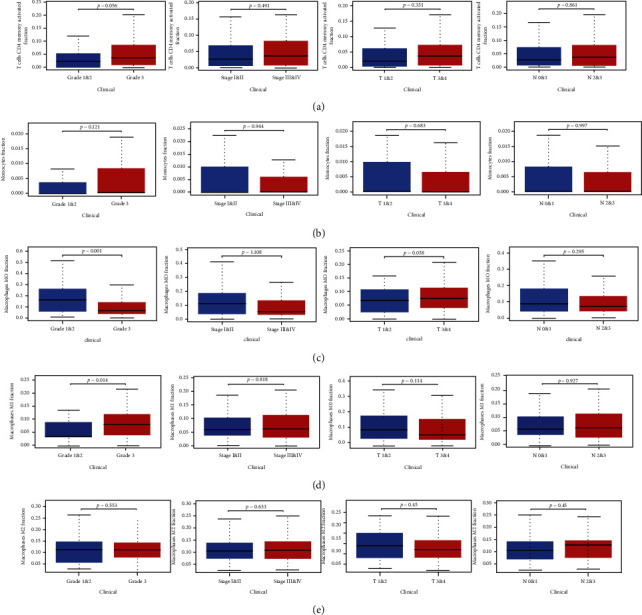
The relationship between the abundance ratio of immune cells and clinical characteristics. (a–e) The relationship between the abundance ratio of each immune cell and tumor grade, clinical stage, T-stage, and N-stage. The upper and lower sides of the boxplot are 75% and 25% quantiles. The line in the middle of the box indicates the median.

According to the clinical characteristics, 153 tumor samples were grouped to analyze the relationship between the abundance of five immune cells and the clinical characteristics (including T-stage, N-stage, clinical stage, and tumor grade), so as to determine the influence of the abundance ratio of immune cells on the clinical characteristics of STAD. As shown in Figure 3, the abundance ratio of macrophages M0 in the high-grade samples (Grades 1 and2) was significantly lower than that in the low-grade group (Grade 3). The abundance of macrophages M1 increased with the increase of tumor grade. Although the difference was not significant, with the increase of tumor grade, clinical stage, T-stage, and N-stage, the abundance of T cell CD4 memory activation and monocytes increased (Figures 3(a) and 3(b)). In addition, the abundance of macrophages M2 in the N2&N3 group was slightly higher than that of the N1&N2 group (Figure 3(e)).

### 3.4. Identification of DEGs Related to Immune Cells

**Figure 4:**
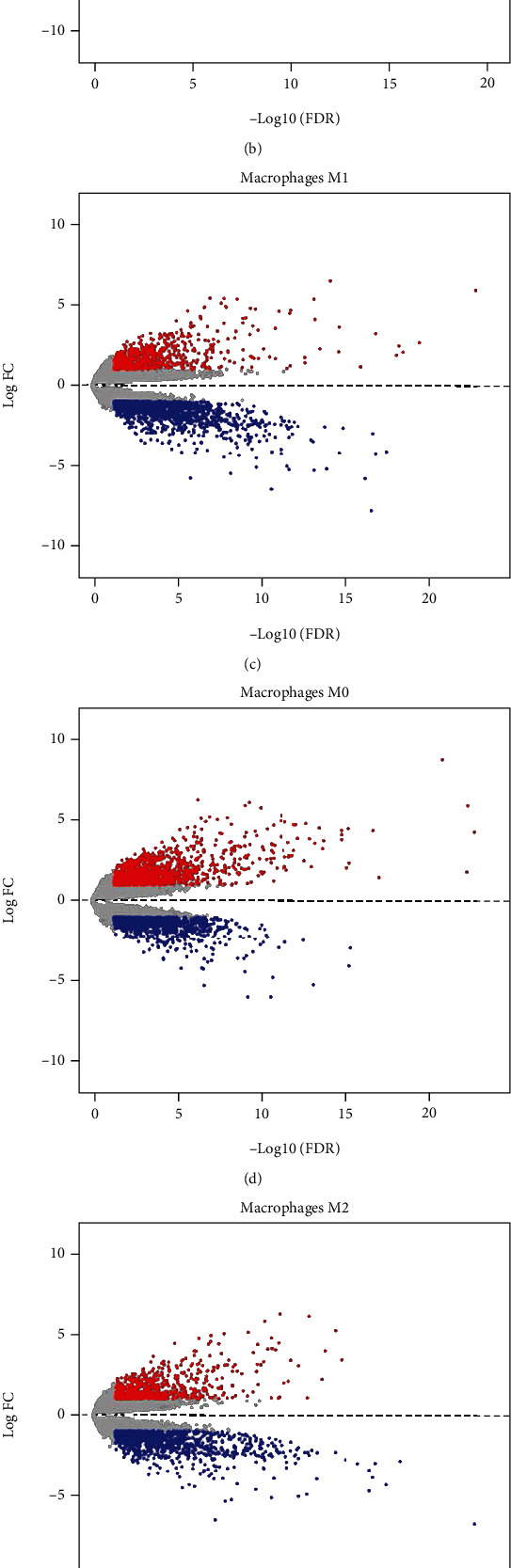
Identification of DEGs associated with immune cells. (a–e) Volcano plots of the GC gene expression profiles grouping by T cell CD4 memory activated, monocytes, macrophages M0, macrophages M1, and macrophages M2. Red represents upregulated genes, blue represents downregulated genes. ∣Log 2 FC | >1 and *P* value < 0.05. (f) Venn calculation results using an online tool to obtain genes involved in the infiltration of five immune cells. The numbers in different color blocks represent the number of genes related to immune cell infiltration. A total of 295 genes are related to the five immune cells.

In order to identify immune-related DEGs, the STAD samples were grouped (high abundance group and low abundance group) according to the median abundance of the five immune cells. We found that there were 475 DEGs in T cell CD4 memory activation, 269 genes with downregulated expression, and 206 genes with upregulated expression. In monocytes, there were 360 DEGs, including 173 genes were downregulated and 187 genes were upregulated. There were 488 DEGs, 139 downregulated genes and 349 upregulated genes in macrophages M0. There were 511 DEGs in macrophages M1, with 313 downregulated genes and 198 upregulated genes. 440 DEGs were identified in macrophages M2, including 272 downregulated genes and 169 upregulated genes. Volcano graphs were used to show the results in Figures 4(a) and 4(e). The Venn diagram analysis shown in Figure 4(f) revealed 295 DEGs related to immune cells.

### 3.5. Enrichment Analysis of Genes Related to Immune Cells

**Figure 5:**
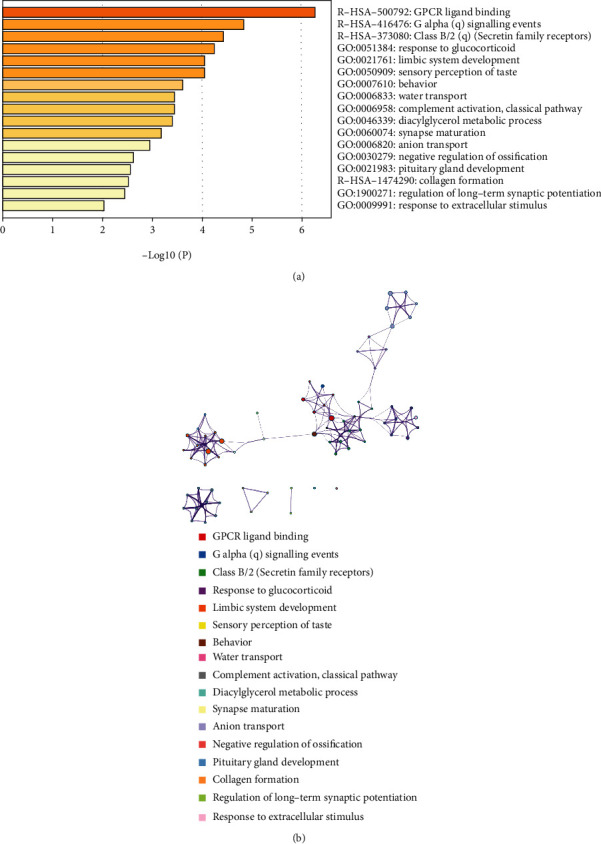
Metascape analysis. (a) Bar graph of enriched terms across input gene lists, colored by *P* values. (b) Network of enriched sets colored by cluster ID, where nodes that share the same cluster ID are typically close to each other.

In order to investigate the functions of the 295 immune-related DEGs, enrichment analysis was performed by using the Metascape. The Metascape analysis shows the top 17 clusters of enriched sets (Figure 5). These genes were enriched in the biological process (BP) categories response to glucocorticoid, limbic system development, sensory perception of taste, and so on. The KEGG pathway data were enriched in GPCR ligand binding, G alpha (q) signaling events, Class B/2 (Secretin family receptors), etc.

### 3.6. PPI Network Construction, Module Analysis, and Identification of Hub Genes

**Figure 6:**
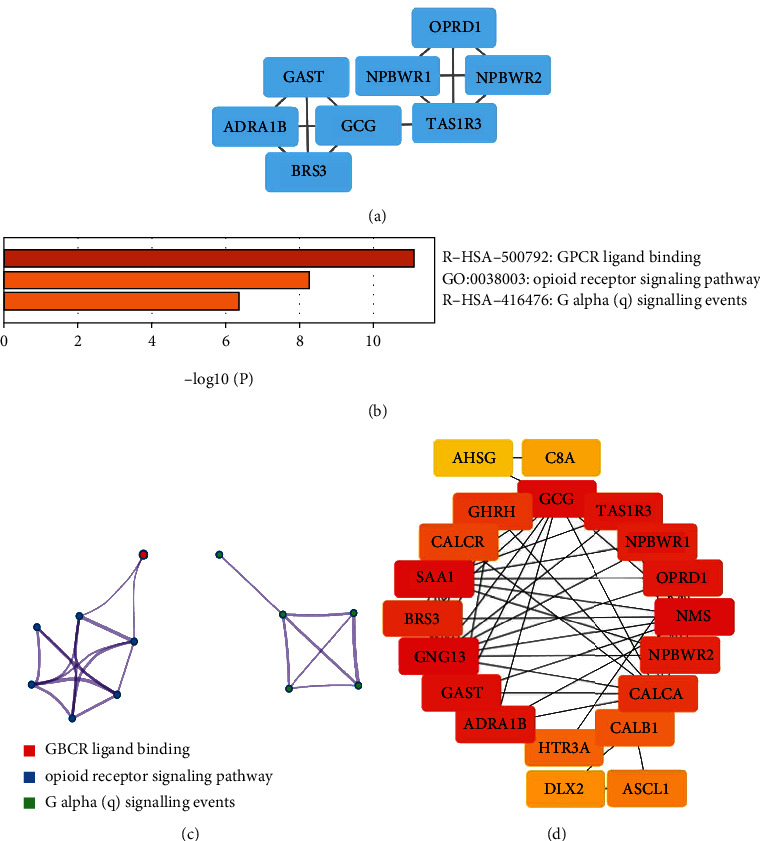
PPI network construction and module analysis. (a) The module with the highest score obtained using the MCODE plugin. (b and c) Metascape analysis. (d) Top 20 genes selected based on MCC methods. The darker the color of the node, the higher the score.

In order to explore the correlation of the 295 immune-related genes and obtain hub genes, PPI and module analysis were conducted. The module with the highest score was shown in Figure 6(a). To explore the function of this module, we conducted an enrichment analysis by using the Metascape and the results showed the genes were enriched in the BP categories opioid receptor signaling pathway. For KEGG pathway, these genes showed enrichment in GPCR ligand binding and G alpha (q) signaling events (Figures 6(b) and 6(c).

The hub genes were determined by the PPI network by using the Cytohubba plugin. The MCC methods were performed to calculate the top 20 genes, which were considered as hub genes, as shown in Figure 6(d).  Table 1 shows the information of 20 hub genes, including complete gene names and main functions.

### 3.7. Survival Analysis of Hub Genes

**Figure 7:**
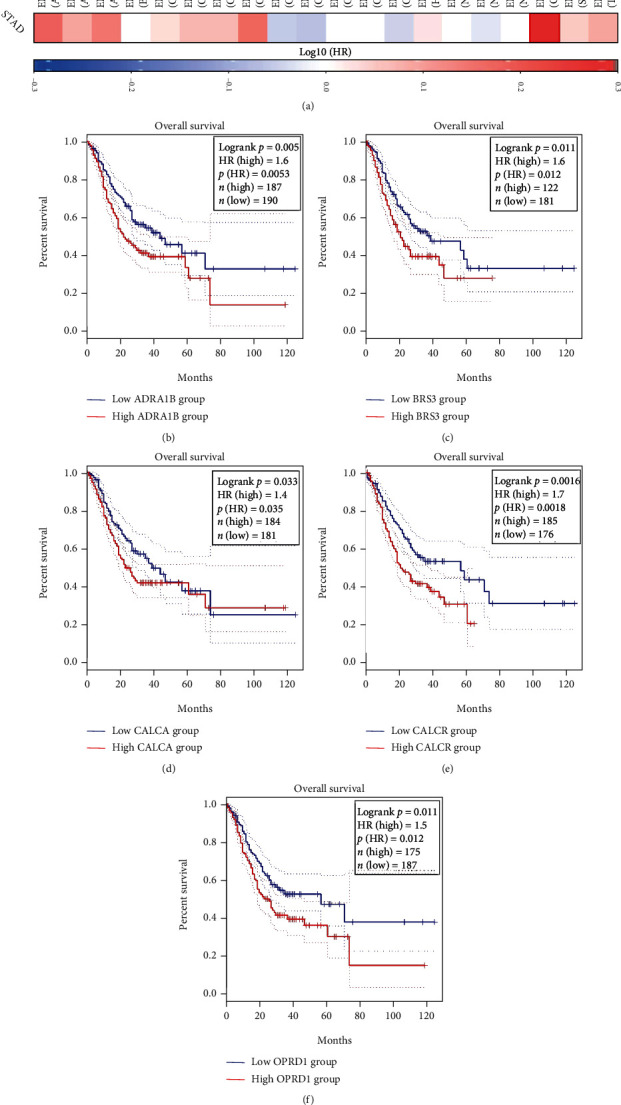
Survival analysis of the hub genes. (a) Survival map of 20 hub genes obtained through the online tool GEPIA2. Red means positive correlation, blue means negative correlation. (b–f) The five genes closely related to survival of STAD. The red line indicates the group with high gene expression, and the blue line indicates the group with low gene expression.

These 20 hub genes are potential immunotherapy targets, and their relationship with prognosis of GC is of great value for further immune-related research.  Figure 7(a) is a survival map of 20 hub genes obtained through the online tool GEPIA2. Figures 7(b)–7(f) show the five hub genes significantly related to overall survival of STAD, namely, ADRA1B, BRS3, CALCA, CALCR, and OPRD1.

### 3.8. Validation of the Five Hub Genes

**Figure 8:**
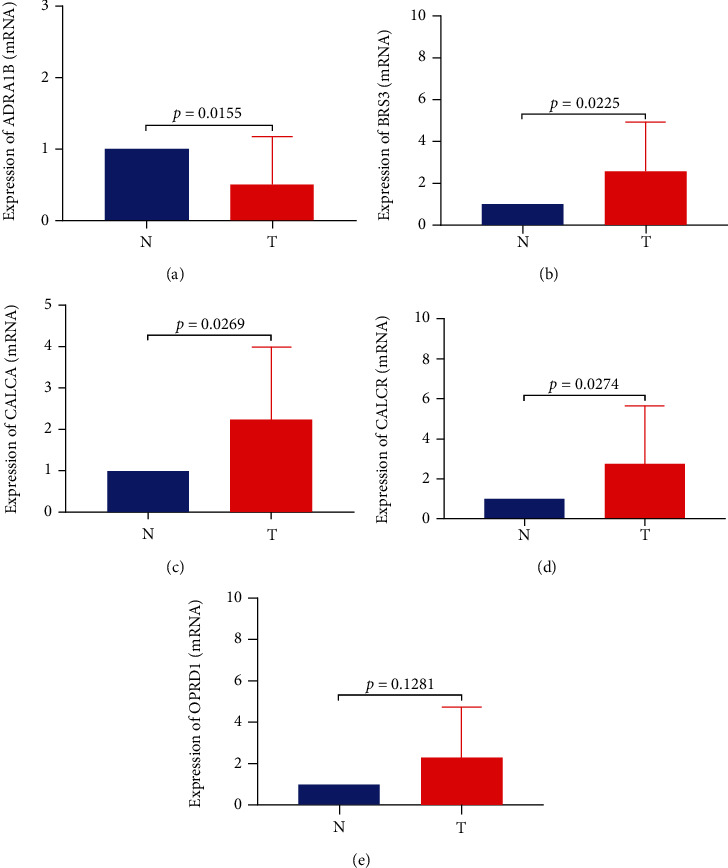
Validation of the five hub genes. (a–e) RT-PCR detected the expression of ADRA1B, BRS3, CALCA, CALCR, and OPRD1 in 18 pairs of cancer and adjacent tissues. *N* represents 18 cases of adjacent tissues, and *T* represents 18 cases of cancerous tissues.

For validating the five hub genes related to survival, we detected the expression difference of these five genes in 18 pairs of cancer and adjacent tissues by RT-PCR, and the results showed that the expression of ADRA1B mRNA in the adjacent tissues was higher than that in the adjacent tissues, while the expression of BRS3, CALCA, and CALCR mRNA in the cancer tissues was significantly higher than that in the adjacent tissues. In addition, the expression of OPRD1 in cancer tissues was also higher than that in adjacent tissues, with an insignificant difference (Figure 8).

## 4. Discussion

GC is the most common malignant tumor in the digestive system. Traditional radiotherapy and chemotherapy are not effective for some patients, so it is imperative to seek new treatment. In recent years, with the development of immunotherapy in multiple cancers, PD-1 inhibitors have received widespread attention in the treatment of GC [22–24]. However, not all patients can get a considerable curative effect, so it is particularly important to look for biomarkers with predictive value and screen the beneficiary population. The purpose of the study was to screen and identify immune cells and genes closely related to immune and clinical outcomes in the STAD microenvironment. This study has not only identified the potential cells and gene targets of STAD immunotherapy but also provided new research ideas for the other tumor immunotherapy.

In the study, we found T cell CD4 memory activated, monocytes, macrophages M0, macrophages M1, and macrophages M2 were highly infiltrated in tumor samples. CD4+ memory T cells play an important role in the occurrence and development of tumors [25]. CD4+ central memory T (TCM) cells maintain immune memory and play a protective role in tumor metastasis [26, 27]. CD4+ effector memory T (TEM) cells express adhesion molecules and chemokine receptors, which perform rapid functions [28]. Both of them play an important role in antitumor immunity. In the peripheral blood of patients with advanced cancer, the proportion of TCM cells decreased and TEM cells increased, showing a typical state of T cell exhaustion [29]. In this study, it was found that the content of T cell CD4 memory activated in tumor tissue was significantly increased. Monocytes appear to be recruited to tumor tissue throughout the tumor progression, including the early stages of tumor growth [30, 31] and the establishment of distant metastasis [32, 33], under the influence of the tumor microenvironment, it can differentiate into tumor-related macrophages, thus promoting tumor growth and metastasis [34]. This study showed that the content of monocytes in tumor samples increased significantly, which further proved the role of monocytes in promoting tumor development. Macrophages can be divided into classic macrophages M1 and alternative macrophages M2 according to their functions [35]. Macrophages M1 is involved in inflammation and antitumor immunity, while macrophages M2 have the characteristics of promoting tumor development [36, 37]. Macrophages M0 are formed by monocytes and have not been polarized to M1 or M2 macrophage subtypes in tumors [38]. In the initial stage of tumor formation, monocytes in peripheral blood gather around the tumor and are mainly polarized to macrophages M1, which plays an antitumor immune role. However, once the tumor has formed, under tumor microenvironment conditions of partial hypoxia and partial acid, macrophages are polarized to M2 type, which has the role of promoting tumor growth, invasion, and angiogenesis and suppressing the immune response [39, 40]. This explains that the infiltration of macrophages M0, M1, and M2 in tumor samples was higher than that in normal samples. In summary, the five cells identified in this study are most likely to play an important role in tumor immune infiltration and GC immunotherapy, confirming the credibility of cell-based immune-related gene analysis.

Enrichment analysis of immune-related genes shows that these genes are mainly related to G protein-coupled receptors (GPCRs) ligand binding. GPCR, which represents the largest gene family in the human genome, plays a vital role in various physiological functions as well as tumor growth and metastasis [41]. Crosstalk between different receptors, including GPCRs, triggers related biological functions of normal and tumor cells [42]. It has been reported that many GPCRs activate many signaling pathways that interact with other plasma membrane receptors [42]. For example, crosstalk between acetylcholine muscarinic receptors (mAChRs), epidermal growth factor (EGFR), and platelet-derived growth factor (PDGFR) receptors leads to the activation of mitotic pathway, which mediates cell proliferation, differentiation, and survival [43]. Some GPCR ligands, such as bradykinin (BK), LPA, gastrin-releasing peptide (GRP), and bombesin (BN), activate EGFR and then induce stimulation in different types of tumors, such as prostate cancer, breast cancer, and pancreatic adenocarcinoma [44] Besides, studies have shown that leukocytes, including neutrophils, T cells, and dendritic cells, mainly perceive signals of movement, migration, chemotaxis, and localization through GPCRs and induce intracellular premigration response through the combination of agonists [45, 46]. These studies indicate that GPCR is closely related to the tumor immune microenvironment.

A total of 20 hub genes were finally identified, five of which were related to survival, namely, ADRA1B, BRS3, CALCA, CALCR, and OPRD1. Studies have shown that these genes are related to the occurrence of certain tumors. ADRA1B is a member of the GPCRs, and it has been reported that this gene is closely related to the prognosis of thyroid papillary carcinoma [47]. Studies have shown that the methylation of ADRA1B plays a key role in the occurrence and development of GC [48, 49]. Our study showed that the expression of ADRA1B in GC tissues was lower than that in adjacent tissues, which may be related to methylation. BRS3 is a G protein-coupled membrane receptor that binds bombesin-like peptides, it is widely distributed in the peripheral tissues and central nervous system, as well as some tumors [50, 51]. It has been reported that BRS3 activation promotes metastasis formation and drug resistance in small cell lung cancer cells [52]. In addition, studies have shown that relative to normal tissue, primary neuroendocrine tumor expression of BRS3 was increased by 13-fold [53]. Similarly, our study also showed that BRS3 expression in GC tissues was significantly higher than that in adjacent tissues. CALCA encodes a peptide hormone that plays a key role in maintaining serum calcium levels and the regulation of T and B cells in some cancers, which are often methylated in many types of cancer [54, 55]. Multiple studies have shown that the level of CALCA methylation in GC tissue is significantly higher than that of normal gastric tissue [56–58]. However, our study suggested that CALCA was highly expressed in cancer tissues, which may need to be further verified by relevant experiments. As a member of GPCRs, CALCR binds to its ligand and calcitonin and regulates a variety of downstream signaling pathways, thus regulating bone metabolism, calcium flux, and cancer cell proliferation [59, 60]. It has been reported that CALCR expression is significantly upregulated in non-small-cell lung cancer and positively correlated with tumor invasion [61]. And another analysis also showed that CALCR is closely linked to the survival of GC, which is consistent with the results of this study [62]. OPRD1 encodes the delta opioid receptor, which is a member of the opioid GPCRs [63], and plays an important role in potassium homeostasis [64, 65] and glucose metabolism [66]. In order to satisfy the needs of rapid proliferation of energy and biosynthesis, tumor cells use aerobic glycolysis to rapidly supply energy [67]. Compared with adjacent tissues, OPRD1 expression was slightly increased in GC tissues in our study. Therefore, there is an energy competition between tumor cells and immune cells, and OPRD1 may act as a regulatory role.

In summary, we identified five immune cells and 20 hub genes, five of which were shown to be related to the overall survival of STAD patients and were significantly associated with some immune cell infiltration. These cells and genes can be considered biomarkers for prognosis as well as markers for STAD therapy, which may be a focus of STAD immunotherapy. However, the evidence from bioinformatics and RT-PCR alone seems to be insufficient, and more relevant experiments such as flow cytometry should be used to verify the results. The potential relationship between tumor microenvironment, STAD immunotherapy, and prognosis can be rerecognized through the in-depth study of these cells and genes.

## Figures and Tables

**Table 1:** Functional roles of the 20 hub genes.

NO.	Gene	Full name	Function
1	ADRA1B	Adrenoceptor alpha 1B	G protein-coupled receptor activity, alpha1-adrenergic receptor activity, and protein binding
2	AHSG	Alpha 2-HS glycoprotein	Cysteine-type endopeptidase inhibitor activity and endopeptidase inhibitor activity
3	ASCL1	Achaete-scute family bHLH transcription factor 1	DNA-binding transcription factor activity and DNA-binding transcription factor activity
4	BRS3	Bombesin receptor subtype 3	G protein-coupled receptor activity and bombesin receptor activity
5	C8A	Complement C8 alpha chain	Encodes the alpha subunit of C8
6	CALB1	Calbindin 1	Calcium ion binding and protein binding
7	CALCA	Calcitonin-related polypeptide alpha	Calcitonin receptor binding and hormone activity
8	CALCR	Calcitonin receptor	G protein-coupled peptide receptor activity and contributes to amylin receptor activity
9	DLX2	Distal-less homeobox 2	DNA-binding transcription activator activity and RNA polymerase II-specific
10	GAST	Gastrin	Hormone activity and protein binding
11	GCG	Glucagon	Glucagon receptor binding and hormone activity
12	GHRH	Growth hormone-releasing hormone	Growth hormone-releasing hormone activity and neuropeptide hormone activity
13	GNG13	G protein subunit gamma 13	G-protein beta-subunit binding and GTPase activity
14	HTR3A	5-hydroxytryptamine receptor 3A	Neurotransmitter receptor activity and protein binding
15	NMS	Neuromedin S	G protein-coupled receptor binding
16	NPBWR1	Neuropeptides B and W receptor 1	G protein-coupled receptor activity and neuropeptide binding
17	NPBWR2	Neuropeptides B and W receptor 2	G protein-coupled receptor signaling pathway and neuropeptide signaling pathway
18	OPRD1	Opioid receptor delta 1	G protein-coupled receptor activity and enkephalin receptor activity
19	SAA1	Serum amyloid A1	G protein-coupled receptor binding and chemoattractant activity
20	TAS1R3	Taste 1 receptor member 3	G protein-coupled receptor activity and signaling receptor activity

## Data Availability

Publicly available datasets were analyzed in this study, these can be found in The Cancer Genome Atlas (https://tcga-data.nci.nih.gov/tcga/).
